# Insufficiency fractures in patients with sacral chordoma treated with high-dose radiation therapy with and without resection

**DOI:** 10.1093/bjro/tzaf001

**Published:** 2025-01-16

**Authors:** Vesna Miladinovic, Robert J P van der Wal, Natasha M Appelman-Dijkstra, Ana Navas Cañete, Wilco C Peul, Johan L Bloem, Augustinus D G Krol

**Affiliations:** Department of Radiation Oncology, Leiden University Medical Center, Leiden 2333ZA, The Netherlands; Department of Radiology, Leiden University Medical Center, Leiden 2333 ZA, The Netherlands; HollandPTC, Delft 1518 JH, The Netherlands; Department of Orthopedic Surgery, Leiden University Medical Center, Leiden 2333 ZA, The Netherlands; Department of Internal Medicine division Endocrinology, Leiden University Medical Center, Leiden 2333 ZA, The Netherlands; Department of Radiology, Leiden University Medical Center, Leiden 2333 ZA, The Netherlands; University Neurosurgical Center Holland, Leiden University Medical Center, Leiden 2333 ZA, The Netherlands; Department of Radiology, Leiden University Medical Center, Leiden 2333 ZA, The Netherlands; Department of Radiation Oncology, Leiden University Medical Center, Leiden 2333ZA, The Netherlands; HollandPTC, Delft 1518 JH, The Netherlands

**Keywords:** insufficiency fractures, sacral chordoma, high-dose radiation, proton beam

## Abstract

**Objectives:**

Determine the incidence, location, and features of insufficiency fractures (IFs) in sacral chordoma patients treated with high-dose radiation therapy (HDR) with(out) resection, relative to radiation therapy type and irradiation plans.

**Methods:**

Clinical data, including details of all surgical procedures and radiotherapies of patients histologically diagnosed with sacral chordoma between 2008 and 2023 available at our database, were retrospectively reviewed. Inclusion criteria were as follows: availability of diagnostic, treatment planning and follow-up magnetic resonance and/or computed tomography scans, and completed treatment. Scans were re-evaluated for the presence and location of IF defined as linear abnormalities with(out) bone marrow oedema (BME)-like changes.

**Results:**

From 48 included patients (29 male, median age 66, range 27-85), 22 were diagnosed with 56 IF (45.8%). IF occurred 3-266 months following the treatment. All sacral and iliac bone IF had vertical components parallel to the SI joint. Twenty patients had bilateral and 16 unilateral IF. BME-like changes were visible in 46 IF (82.1%, 0.80, *P* ≤ .001). In 13/56 IF (23.2%), BME-like changes were seen prior to IF diagnosis; in only 1 patient, BME-like changes did not develop into an IF. Thirty-nine IF (84.7%) occurred within low-dose volume and 7 (15.3%) outside of irradiated volume in 16/44 irradiated patients. Six IF occurred in 1 patient treated with surgery only.

**Conclusions:**

Pelvic IFs are common in sacral chordoma patients treated with definitive or (neo)adjuvant HDR, occurring months to years following treatment. Not all IF occur in the irradiated volume.

**Advances in knowledge:**

When present, BME-like changes indicate risk of IF developing. IF do not heal over time.

## Introduction

Chordomas are locally aggressive but rarely metastasizing malignant bone neoplasms that most commonly occur in the axial skeleton, especially in the sacral region. *En bloc* resection, considered as standard treatment of sacral chordoma, is not always feasible due to the anatomical location of the tumour and its functional (neurological deficit) or surgical (high complication rate) outcome. Definitive proton, carbon ion, or photon therapy is therefore often used instead of surgery or as an (neo-)adjuvant treatment to surgery given its benefit in improvement of local control.^1^ Relative resistance of chordomas to irradiation requires application of high-dose radiation therapy (HDR), which is not always possible due to the proximity of organs sensitive for acute or late radiation effects. Already known late effects of HDR in pelvic tumours are insufficiency fractures (IFs) of the pelvis.^2–14^ IF can be asymptomatic; however, if symptomatic, typical complaints include lower back or hip pain and reduced mobility.^2^ Diagnosis is based on bone scintigraphy, magnetic resonance imaging (MRI), or computed tomography (CT).^15^ The diagnosis can be missed because of a low index of clinical and/or radiological suspicion, especially in the absence of clinical symptoms, or because the fracture lies outside of the MRI field of view. Furthermore, a commonly reported pitfall of imaging is that IF can be misinterpreted as metastasis, resulting in unnecessary biopsies and even additional radiotherapy.^16–19^ Consequently, reported incidences of IF following HDR vary largely—from 3% to 52%.^1^^,^^20^

Our aim is to determine the incidence and location of IF in patients with sacral chordoma treated with HDR only or in combination with surgery relative to the radiation therapy types and irradiation plans.

## Methods

Electronic health records of patients histologically diagnosed with chordoma of the sacral region in the past 15 years (April 2008—April 2023), available at our tertiary bone tumour centre database of the Leiden University Medical Center (LUMC), were retrospectively reviewed. The study was approved by the local medical ethics committee, and written informed consent was obtained from all included patients. Inclusion criteria were availability of pre-treatment, treatment planning and treatment follow-up MR and/or CT scans, and completed HDR (proton, carbon ion, or photon) and/or surgery. Scans were re-evaluated by an experienced musculoskeletal radiologist (*A.N.C.*) for the presence and exact location of the IFs. IF were defined on MRI scans as clear linear abnormalities, with or without accompanying bone marrow oedema (BME)-like changes, without an associated tumour mass.^16^^,^^17^ Bone marrow oedema-like changes were identified and localized on fluid-sensitive MR sequences as ill-defined areas of increased signal intensity without cortical destruction and/or associated soft tissue mass.

### Scanning protocols

Pre-treatment and follow-up MRI was performed on 1.5 T or 3.0 T scanners (Ingenia, Philips, Best, Netherlands) according to the following protocol: survey turbo field echo (TFE); T1-weighted turbo spin echo (TSE), with echo times (TE 10-20 ms) and repetition times (TR 600-700 ms); T2-weighted TSE mDixon with TE (60-80 ms), TR (2500-5000 ms), and with water, fat, and in-phase reconstructions; dynamic sequence including T1 map TFE; Gd-chelate enhanced fat SPIR T1-weighted TSE, TE (10-20 ms), and TR (600-700 ms). CT scans (Toshiba, Aquilion ONE, Tokyo, Japan) were performed using a 120 kVp energy setting, 3-mm slice thickness, and 0.5 s rotation time.

### Radiotherapy planning and contouring

Contouring for proton therapy was done according to the SACRO protocol guidelines.^21^ Macroscopic tumour volume visible on pre-treatment CT and MRI was contoured as high-dose volume (gross tumour volume—GTV), whereas areas with possible microscopic spread were segmented as an elective treatment volume (clinical target volume—CTV). Doses of 74 GyE and 59.2 GyE were administrated to the GTV and CTV, respectively, in 37 fractions over the 7-week treatment period. Treatment was robustly optimized considering 3% density uncertainty along 3 axes resulting in 21 dose optimization scenarios and 28 dose evaluation scenarios. Evaluation was based on the work of Korevaar *et al.*^22^

Carbon ion therapy to a dose of 60 or 64 GyE was delivered in a hypofractionated schedule of 20 or 16 fractions, respectively, over 4 weeks. The high-dose volume (GTV) was contoured on the pre-treatment CT and MRI, to which a 2 cm margin was added to encounter for the surrounding microscopic areas of the tumour (CTV). The low-dose area (planning target volume—PTV) was defined with 7 mm additional margin to the high-dose volume (GTV + CTV) to account for all the treatment setup uncertainties. Data on irradiated volume of patients treated with carbon ion therapy was not available as the patients were irradiated elsewhere.

Photon therapy total high-dose of 50-60 Gy was administrated in 25-30 fractions, 2 GyE per fraction, over 5-7 weeks. Target volume contouring was based on pre-treatment CT scans. The low-dose volume (PTV) was contoured with a 1.5-2 cm margin from the high-dose volumes (GTV and CTV). For PTV receiving ≥95% of its prescribed dose (V95%), coverage aim was ≥99% and constraint ≥98%. For PTV dose to 0.03 cm^3^ volume (D0.03), constraint was ≤107%. For cauda equina (D0.03) constraint was <60%; for rectum (V30Gy) constraint was ≤70%, for rectum (V40Gy) constraint was ≤60%, for rectum (V50Gy) constraint was ≤30%, and for rectum (V61Gy) constraint was ≤5%. All patients were treated in the supine position.

### Clinical data and follow-up

Magnetic resonance imaging was performed at inclusion and every 6-7 months during the follow-up in addition to more frequent clinical evaluation; on average, 8 scans in total were made in each patient. Clinical data, including gender, age, presence of symptoms (pain and/or reduced mobility), body mass index (BMI), previous diagnosis of osteoporosis and menopause, as well as details of all surgical procedures and radiation therapies, were collected from the patient files. Time between the first irradiation or first surgery (in case of multiple surgeries) and diagnosis of the IF on an MR scan was calculated in months for each patient. The location of the IF was compared with both surgical reports and radiotherapy plans to find a potential correlation between IF and treatment, including level of resection and irradiated volumes.

### Statistical analysis

Statistical analysis was performed in RStudio (RStudio PBC, Boston, MA, USA, Version 1.4.1717). Data normality was inspected using Shapiro–Wilk normality test with α = .05 level of significance. The difference of median BMI values of patients with and without IF was evaluated with the *t*-test (α = .05). Correlation matrix (α = .05) between presence of BME, tumour volume, BMI, administrated dose, irradiated volume, number of irradiation fractions (fx) and diagnosis of IF in a patient was computed with *Spearman* method.

## Results

Of the 59 sacral chordoma patients who were retrieved, 48 were included. Eleven were excluded due to incomplete follow-up data (9 patients) or because treatment was not completed at the time of data collection (2 patients). Median follow-up time was 49 months (range 3-421 months). Twenty patients had HDR only, 24 had HDR followed by resection with or without additional adjuvant HDR, and 4 patients had resection only as treatment of the chordoma (Table 1).

**Table 1.** Clinical data and frequency of insufficiency fracture diagnosis among the 48 sacral sarcoma patients.

	Without insufficiency fracture			With insufficiency fracture		
	Male (*n* = 18)	Female (*n* = 8)	Combined (*n* = 26)	Male (*n* = 11)	Female (*n* = 11)	Combined (*n* = 22)
Median age (range)	60	66	64.5	65	69	66
	(29-78)	(46-79)	(29-79)	(27-80)	(43-85)	(27-85)
Primary disease	14	6	20	10	8	18
Recurrent disease	4	2	6	1	3	4
Bone oedema visible on MRI	1	0	1	8	9	17
Osteoporosis treatment	0	0	0	1	3	4
Postmenopausal	*NA*	8	8	*NA*	10	10
Median BMI	25.71	23.62	25.53	28.53	26.24	28.40

Abbreviations: BMI = body mass index; MRI = magnetic resonance imaging; *n* = number of patients; *NA* = not applicable.

**Figure 1.**
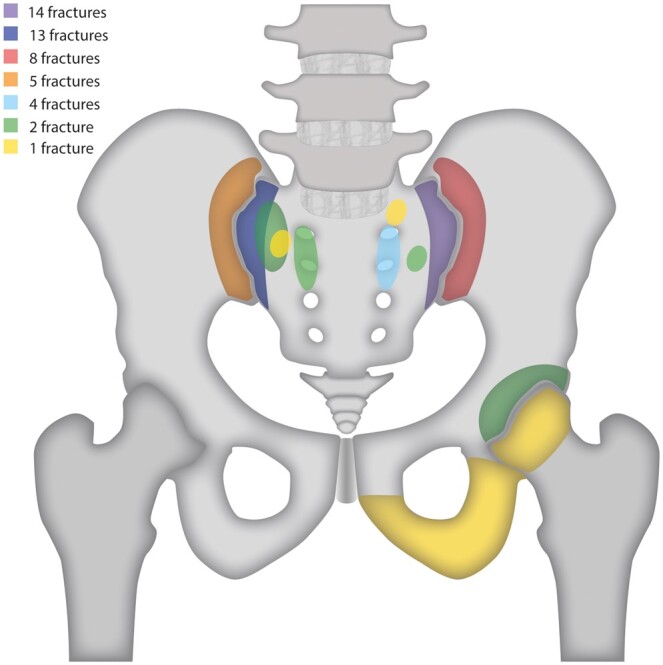
Graphical presentation of frequency distribution of 56 IF in sacral chordoma patients treated with HDR or HDR and surgery. Twenty patients had bilateral and 16 had unilateral IF. Number of fractures within each area is portrayed by different colours as given in the legend. Anatomical area marked with purple corresponding to 14 IF, dark blue area to 13 IF, red area to 8 IF, orange area to 5 IF, light blue area to 4 IF, including 4 different green anatomical areas corresponding to 2 fractures each, and 4 different yellow anatomical areas to 1 IF each, give together 56 IF. IF = insufficiency fractures; HDR = high-dose radiation.

Based on MR, 56 fractures were identified in 22 patients (45.8%). Bone marrow oedema-like changes were visible in 46 of these fractures (82.1%) in 17 patients (77.3%). Bone marrow oedema-like changes were also visible in the femoral head in 1 patient without a fracture line, which developed into osteoarthritis of the hip as diagnosed on MR 46 months later. All sacral and iliac bone IF had vertical components that were parallel to the SI joint. Twenty patients had bilateral and 16 had unilateral IF. Four out of all 56 IF, seen in 3 different patients, occurred outside the sacral-iliac bone region. Frequency of IF per location is presented in Figure 1.

**Figure 2.**
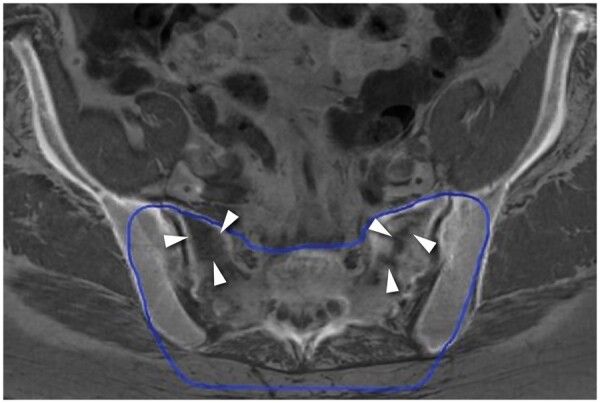
Superimposed MR scan and treatment planning CT scan of a patient with IF (white arrowheads) occurring within the low-dose volume (blue line). CT = computed tomography; IF = insufficiency fractures; MR = magnetic resonance.

Among patients treated with HDR, 4 fractures were found in 2 patients treated with C-ion therapy of whom no details on dose volumes were available as mentioned earlier in the materials and methods section. There were no fractures in high-dose areas as high dose was only given on the tumour mass itself. Thirty fractures occurred in 11 out of 20 patients treated with HDR only, and 20 fractures occurred in 10 out of 22 patients treated with HDR and resection. Thirty-nine IF (84.7%) in 16 patients occurred within the low-dose volume (Figure 2), and 7 fractures (15.3%) in 3 patients occurred outside of the irradiated volume. Six fractures (2 sacral, 2 in the iliac bone, 2 in SI joint) were found in 1 male patient who had a resection (R1) only.

Median age at IF diagnosis was 66 years (range 27-85 years). The frequency of IF in combination with clinical and radiological data are presented in Table 1. Three female and 1 male patients were already prescribed with anti-osteoporosis medication prior to the IF diagnosis, out of which only 1 female patient was previously diagnosed with osteoporosis at the referring hospital. Eighteen female patients (94.7%) were postmenopausal, 10 of whom had IF. There was no statistically significant difference between median BMI values of patients with and without IF (*P* = .899).

The frequency of IF and time to their occurrence relative to therapy given is presented in Table 2. Highest dose administered with photon, carbon ion, or proton therapy was 60 Gy, 64 GyE, and 74 GyE, respectively. Median number of fractions was 36 (range 14-37). The most cranial resection level was at osseous S2 level. Four patients had 2 surgeries and 1 patient had 4 surgeries prior to IF occurrence. Complete tumour resection (R0) was achieved in 5 patients (29.4%) without and in 2 patients (18.2%) with IF. A microscopically positive resection margin (R1) was obtained in 8 patients (47.1%) without and in 7 patients (63%) with IF. Anticipated intralesional resection (R2) was performed in 4 patients (23.5%) without and in 2 patients (18.2%) with IF.

**Table 2.** Frequency of insufficiency fractures (IF) per treatment type and median time (in months) to IF diagnosis following the first high-dose irradiation fraction and the first resection or stabilization surgery.

		Without insufficiency fracture				With insufficiency fracture					
Type of therapy		Number of patients (*n* = 26)	Median dose administrated	Low-dose irradiation volume median value (range) (cm^3^)	High-dose irradiation volume median value (range) (cm^3^)	Number of patients (*n* = 22)	Median dose administrated	Low-dose irradiation volume median value (range) (cm^3^)	High-dose irradiation volume median value (range) (cm^3^)	Median time to IF appearance following surgery (months)	Median time to IF appearance following first fraction (months)
Photon therapy		1	44 Gy	2862	1595.97	0	*NA*	*NA*	*NA*	*NA*	*NA*
Proton therapy		7	74 GyE	1503 (1105-4459)	1044.16 (119.91-3479.69)	10	74 GyE	1236.25 (880.84-2504.18)	272.40 (46.91-1058.84)	*NA*	6 (3-266)
Carbon ion therapy		1	64 GyE	*NA*	*NA*	1^a^	64 GyE	*NA*	*NA*	*NA*	8
Resection +	Photon therapy	6	60 Gy	811 (401-2018)	99.87 (33-197)	6	63.75 Gy	616.41 (268.85-1193.46)	84.78 (51.260-378.284)	27 (5-309)	22 (4-84)
	Proton therapy	6^b^	74 GyE	601 (588-1040)	382 (91-1540)	3^c^	74 GyE	1614.93 (365.13-1818.36)	354.35 (8.14-1391.52)	28 (7-40)	25 (10-25)
	C-ion therapy	2	62 GyE	*NA*	*NA*	1^a^	64 GyE	*NA*	*NA*	65	53
Resection only		3	*NA*	*NA*	*NA*	1	*NA*	*NA*	*NA*	2	*NA*

Abbreviations: *n* = number of patients; Gy = Gray; GyE = Gray equivalent.

a Stabilization surgery for IF.

b 2 patients received combined treatment: 50 GyE proton therapy + resection + 24GyE proton therapy.

c 1 patient received combined treatment: 50 GyE proton therapy + resection + 24GyE proton therapy.

During the follow-up time, none of the fractures healed spontaneously. In 2 patients, each with 1 fracture, surrounding BME-like changes disappeared after 17 and 34 months, but the fracture lines remained. In the other 15 patients, presence of 42 fractures and accompanying BME-like changes persisted. In only 5 patients, with a total of 13 fractures, BME-like changes were visible 7-21 months prior to visible IF line on an MRI.

**Figure 3.**
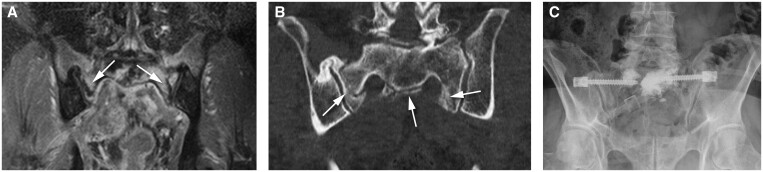
Bilateral sacral IF visible (*arrows*) on T1W SPIR Gd-enhanced MRI (A) and on CT scan (B) with a *“H”*-shape, with a vertical component parallel to the articular surface of the SI joint and a continuous transversal component that crosses the sacrum at the level of S1, prior to the stabilization surgery; (C) radiograph following the stabilization surgery using cementoplasty with placement of screws crossing the SI joint as well. CT = computed tomography; IF = insufficiency fractures; MRI = magnetic resonance imaging; SI = sacroiliac.

**Figure 4.**
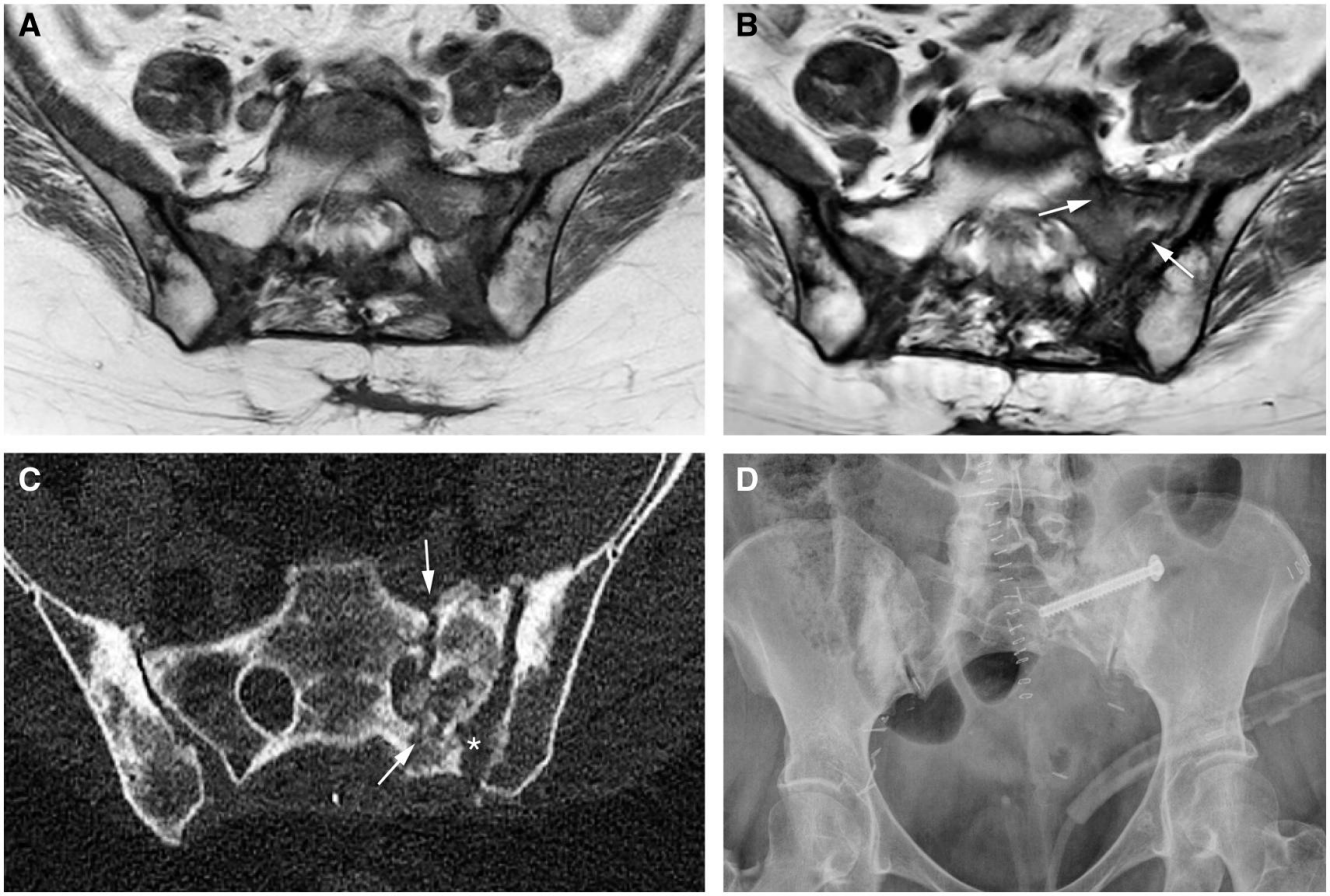
(A) Presence of BME-like changes but no clear fracture line on the left side of the sacrum is visible on the T1W MRI; (B) 18 months later, first appearance of the fracture line (*arrows*) on the T1W MRI; (C) CT scan prior to the stabilization surgery showing a complete fracture line in left part of the sacrum (*arrow*), a small fracture fragment (long *arrow*) is also seen with involvement of the neuroforamen S1 to the left. Diastasis in the left SI joint (*asterisk*) was also observed; (D) radiograph made 7 months following stabilization surgery showing the placement of a screw crossing the SI joint. BME = bone marrow oedema; CT= computed tomography; MRI = magnetic resonance imaging; SI = sacroiliac.

Two patients underwent stabilization surgery due to persistent pain and reduced mobility complaints related to IF. The first patient had pain and bilateral IF without BME-like changes of the sacrum. Symptoms disappeared upon stabilization surgery where 2 screws with cement augmentation were placed at the IF location for sacroiliac joint stabilization (Figure 3). The second patient also had pain and a unilateral IF on the left side of the sacrum. Within this area, 18 months prior to the fracture line appearance, a large area of BME-like change was visible on MRI. Symptoms disappeared after stabilization surgery consisting of decompression of the S1 nerve root and placement of a fixating screw at the fracture side and sacroiliac joint. Bone marrow oedema was still visible on the MRI following the surgery (Figure 4), probably partly also secondary to postsurgical changes.

Only 1 patient was irradiated 2 times, initially with 50 Gy photon radiotherapy and 3 years later re-irradiated with 60 GyE carbon ion therapy. Based on the experience of the treatment centre, re-irradiation dose plan was adapted to a lower total dose of 60 GyE, and number of fraction was increased to 20. No signs of IF fractures were observed during the 5-year follow-up with regular MRI scans performed according to the protocol. Another patient had 77 Gy photon radiotherapy for prostate cancer in close proximity to the resection area of a sacral chordoma. No signs of IF fractures were observed during a 3-year follow-up period with regular MRI scans. None of the patients within this cohort had regional or pulmonary metastasis.

**Figure 5.**
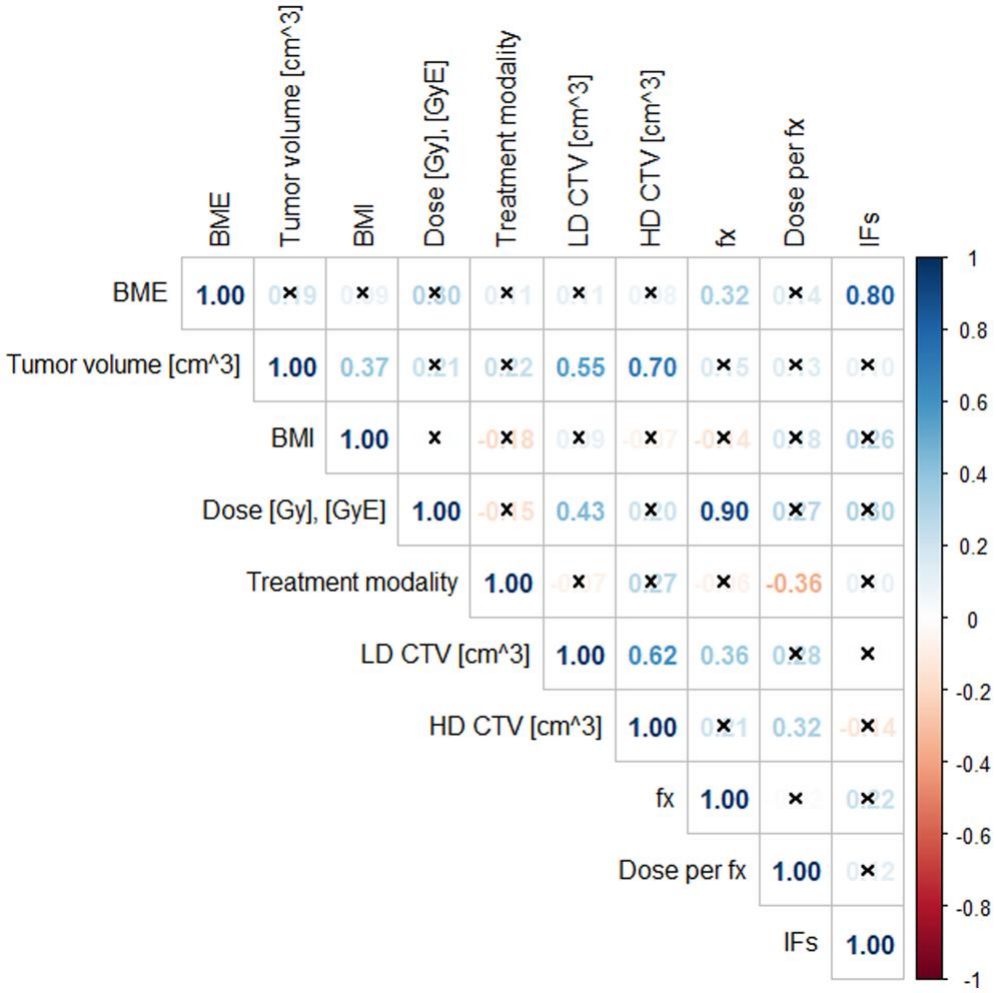
Correlation factors between presence of BME, tumour volume (cm^3^), BMI, administrated dose (Gy, GyE), treatment modality, low- and high-dose CTV (cm^3^), number of fractions (fx), dose per fraction (Gy, GyE), and presence of IF in a patient computed with *Spearman* method (α = .05). (x—statistically not significant correlation factors). BME = bone marrow oedema; CTV = clinical target volume.

The correlation matrix revealed a positive correlation between diagnosis of IF in a patient and tumour volume, BME-like changes, administrated dose, BMI, number of fractions, dose per fraction, and treatment type with factors of 0.10 (*P* = .557), 0.80 (*P* ≤ .001), 0.30 (*P* = .064), 0.26 (*P* = .114), 0.22 (*P* = .173), 0.12 (*P* = .479), and 0.10 (*P* = .547), respectively. However, correlation between IF incidence and BME-like changes was the only statistically significant one. Correlation factors between IF incidence and high-dose CTV and low-dose CTV were −0.14 (*P* = .391) and 0.01 (*P* = .956), respectively (Figure 5).

## Discussion

Pelvic IF’s were common (45.8%) in our patients with sacral chordoma treated with HDR with or without resection or resection only. Although the frequency of IF differed per HDR treatment type, administrated dose, number of fractions, and dose per fraction, these differences did not reach statistical significance. To the best of our knowledge, this is, so far, the only study to report on IF incidence and location, relative to the dose volumes in sacral chordomas treated with HDR and/or tumour resection. Remarkable is the high occurrence of IF in low-dose regions (39/46 fractures, 84.7%) compared to the occurrence outside the irradiated tissue (7/46 fractures, 15.3%), including the occurrence in only 1 out of 4 patients treated with surgery only (6 fractures). Also, the irradiated volumes were not smaller in patients without IF (Table 2). Explanation of the relationship of IF with dose distribution, irradiated volume, and surgery is still elusive, but it is clear that change in loading mechanisms following treatment plays an important role. The reported incidence of IF among sacral chordoma patients treated with definitive or (neo)adjuvant HDR was higher (up to 52%) compared to the IF incidence reported among patients with gynaecological or prostate cancer treated with radiation therapy (up to 33.3%).^1^^,^^20^^,^^23^ As a comparable median dose of 40-50 Gy was administrated to the pelvic area in these patient groups, this suggests that IF incidence is not entirely related to the administrated dose. This is also illustrated by a patient who did not develop an IF during a 5-year follow-up period, despite being irradiated twice within a short period of time (3 years). Also, among gynaecological patients treated with radiation therapy, no pelvic bone dose constraints have been reported to be significant in reduction of IF risk.^24^ But for greater certainty, this correlation between dose distribution and IF frequency and location should be investigated on larger cohorts.

Although IF can be seen already after several months (median 6, range 3-266 months) following the first HDR fraction, they can also occur years later (Table 2). A comparable median time of occurrence (11-36 months) was reported in cervical cancer patients treated with radiation therapy.^3^^,^^16^^,^^23^^,^^25–28^ When HDR is combined with surgery, IF tend to occur later with a median of 22-65 months (range 4-309) (Table 2) regardless of the resection margin (R0, R1, R2). So far, no explanation has been found for these differences.

In all IFs of the sacrum and iliac bones, at least 1 component of the fracture line was vertical and parallel to the SI joint. This is consistent with other reports on IFs in this area where the load of the upper body is transferred to the lower part.^29^^,^^30^ Furthermore, the higher incidence of IFs in the sacrum compared to the iliac bone is related to the greater bone density of the latter.^31^ The majority of IFs (80.4%) were accompanied by BME-like changes as previously reported.^16^^,^^17^^,^^24^^,^^32^ A statistically significant correlation (0.8, *P* ≤ .001) was found between BME-like changes and IF incidence in our cohort. Only 1 patient had BME-like changes without a fracture. This patient had BME-like changes in the femoral head 46 months prior to diagnosis of osteoarthritis in the hip joint. The presence of BME-like changes, appearing prior to the visibility of an IF was relatively rare, and occurred 7-21 months before only in 13 fractures (23.2%). Although relatively infrequent, the presence of BME should raise the suspicion of imminent IF, as we encountered BME-like changes without IF in only 1 patient.

High BMI is often reported as a potential risk factor for development of IF following radiotherapy; however, this was not the case in our patients.^17^^,^^24–28^^,^^33^^,^^34^ Thus, BMI cannot be used as a warning sign of impeding IF in patients with characteristics similar to ours.

None of the IFs encountered in this cohort have healed spontaneously during the follow-up time, nor, to our knowledge, has such an occurrence been reported before. Two of our patients responded with markedly reduced pain after surgical fixation of the IF. Although pain may be associated with the primary tumour, and IF can be asymptomatic, the presence of IF should be taken into consideration as a cause of pain.

Lately, more attention has been given to irradiation-induced IF and their prevention and treatment among cervical and prostate cancer patients using pharmacological interventions, as IF, accompanying pain and reduced mobility, can have a negative impact on patient’s quality of life.^28^^,^^35^ Analogous attention should be given to sacral chordoma patients treated with HDR and/or resection. Prevention or treatment of IF in a non-invasive way, such as use of bisphosphonates, would impede additional (stabilization) surgeries, IF-caused pain, and reduction of mobility.^31^^,^^36^

There are several limitations to this study, mainly related to its retrospective nature. Although the MR studies were not obtained on fixed time points, as would have been the case in a prospective study, the median time interval between an MR study showing an IF and the preceding MRI was 7 months. This means we could have missed a BME-only phase preceding an IF. We did not have the data to assess the potential role of osteoporosis. As no dual X-ray absorptiometry scans were available for patients of this cohort, no analysis on bone mineral density prior to and following the radiation therapy could be performed, which could have potentially given a better insight into the degree of involvement of radiation dose in IF incidence. Further, no reliable data on reported patient pain was available due to the retrospective nature of the study. Because of the rarity of this kind of tumour, and the various therapeutic approaches, numbers are small.

## Conclusion

Pelvic IFs are common (45.8%) in patients with sacral chordoma treated with definitive or (neo)adjuvant HDR, or surgery only and are not dose distribution related. They can occur months to years after the first fraction of HDR and do not heal over time. In the majority of cases (82.1%), IFs are accompanied by BME-like changes, and these can be a sign of imminent IF.
